# Crossing the pond: genetic assignment detects lobster hybridisation

**DOI:** 10.1038/s41598-020-64692-z

**Published:** 2020-05-08

**Authors:** Charlie D. Ellis, Tom L. Jenkins, Linda Svanberg, Susanne P. Eriksson, Jamie R. Stevens

**Affiliations:** 1 Hatherly Laboratories, Department of Biosciences, College of Life and Environmental Sciences, University of Exeter, Exeter, EX4 4PS UK; 2 Kristineberg Marine Research Station, Department of Biological and Environmental Sciences, University of Gothenburg, Kristineberg 566, 45178 Fiskebäckskil, Sweden

**Keywords:** Ecology, Evolution, Genetics, Molecular biology

## Abstract

American lobsters (*Homarus americanus*) imported live into Europe as a seafood commodity have occasionally been released or escaped into the wild, within the range of an allopatric congener, the European lobster (*H. gammarus*). In addition to disease and competition, introduced lobsters threaten native populations through hybridisation, but morphological discriminants used for species identification are unable to discern hybrids, so molecular methods are required. We tested an array of 79 single nucleotide polymorphisms (SNPs) for their utility to distinguish 1,308 *H. gammarus* from 38 *H. americanus* and 30 hybrid offspring from an American female captured in Sweden. These loci provide powerful species assignment in *Homarus*, enabling the robust identification of hybrid and American individuals among a survey of European stock. Moreover, a subset panel of the 12 most powerful SNPs is sufficient to separate the two pure species, even when tissues have been cooked, and can detect the introduced component of hybrids. We conclude that these SNP loci can unambiguously identify hybrid lobsters that may be undetectable via basic morphology, and offer a valuable tool to investigate the prevalence of cryptic hybridisation in the wild. Such investigations are required to properly evaluate the potential for introgression of alien genes into European lobster populations.

## INTRODUCTION

Genetic introgression due to hybridisation with non-native species is a major consequence of human-facilitated introductions that threatens endemic species with reduced fitness and local replacement^1,2^. Even where hybridisation between native and introduced species is rare, rapid and extensive genetic introgression can arise^3^. Population-level introgression and genetic admixture are not necessarily undesirable—managed interbreeding has been proposed to enhance resilience in key species threatened with extinction^4^—but invasive hybridisation is often associated with profoundly harmful effects, including loss of genetic variation and adaptation^1,5^. Nevertheless, assessing the conservation threat posed by hybridisation is not straightforward, and is especially challenging when hybrids themselves are fundamentally difficult to identify^5,6^. Investigation of hybridisation and management of its impacts require tools to distinguish hybrids from pure species strains, and the increasing availability of molecular markers presents a powerful resource to assess the extent of crossbreeding and introgression in wild populations subject to introductions^7,8^.

The European lobster (*Homarus gammarus*) is renowned for its high value as a seafood commodity, but stock collapses have severely diminished the productivity of fisheries throughout extensive portions of the species’ range^9^. In Europe, recent annual landings of *H. gammarus* of ~5,000 tonnes are dwarfed by those of its transatlantic congener, the American lobster (*H. americanus*), which supports vast harvests of >150,000 tonnes per year^10^. To satisfy European demand for lobster that native *H. gammarus* landings cannot fulfil, ~17,000 tonnes of whole American lobsters are imported annually, around three quarters of which are imported live^11^, with the remainder comprised mainly of whole animals which are shipped frozen having already been cooked^12,13^.

Perhaps inevitably, the import of live *H. americanus* has led to their introduction into European waters via their escape or release^14,15^. In one high-profile case from 2015, some 361 American lobsters were released during a religious ceremony in Brighton, UK, with some introduced females later recovered bearing fertilised eggs and hundreds more never recovered at all^16^. Lobsters captured in European habitats and reported as *H. americanus* by fishers, managers or scientists have led to concerns that its presence poses a threat to native species, especially *H. gammarus*^14,17^. Citing their potential for invasiveness and to threaten native lobsters via competition and genetic introgression, Norway banned the import of live *H. americanus* in 2016, while the same concerns led Swedish officials to launch a bid, ultimately unsuccessful, to add the species to the European Union’s list of invasive alien species^11^, which would have prohibited the live trade of American lobsters throughout the EU bloc.

Although concerns that pathogenic syndromes of *H. americanus* may spread to devastate *H. gammarus* stocks have been tempered by the latter’s apparent disease resilience^18,19^, fears that American lobster introductions may impact European populations through competition and genetic introgression have not been so readily mitigated, particularly following recent observations that Americans both predate on and interbreed with European counterparts^20^. Reports of wild hybridisation are concerning as well as surprising; while long established that *Homarus* hybrids could be bred in captivity through induced fertilisation^21^, it was also proposed that behavioural characteristics of sexual selection should constrain interspecific mating in the wild^22^. However, this appears not to be the case^20^. Molecular identification of suspected *Homarus* hybrids has to date been reliant on the microsatellite method of Jørstad *et al*. (^23^, cited in^20^), though neither the sequences nor assignment power of these three loci have ever been published. Reliance on so few loci can also limit power for the detection of hybridisation and introgression following backcrossing^8^.

Most lobsters reported as alien *H. americanus* in Europe are taxonomically identified on the basis of heterospecific morphological indicators, especially differences in exoskeleton pigmentation and the presence of a spine on the ventral surface of the rostrum that is absent in *H. gammarus*^23,24^. Hybrids, however, cannot be visually identified since they may display characteristics of either parent or a mixture of both, and these methods also have limitations in distinguishing the pure species; the ventral rostral spine is unreliable, with both species capable of displaying the supposedly diagnostic characteristic of the other, and exoskeletal colouration can vary broadly^14,15,23^. Moreover, colouration is effectively invalidated as a determinant by cooking, which turns the shell of both species red. This presents another means by which *H. americanus* may impact *H. gammarus* as a seafood commodity, albeit through economic, rather than ecological, competition. In the UK, a 400 g cooked native European lobster typically costs in excess of £20^25^, whereas an equivalent *H. americanus* imported from Canada retails at about half this price (e.g. £11^26^). Given this difference in value, the limitations of morphologically diagnostic characteristics present clear opportunity for exploitative mislabelling^27^, a widespread traceability issue impacting seafood supply chains.

The development of modern molecular resources is required to enable comprehensive assessments of the potential for *H. americanus* to threaten its congener in Europe through hybridisation in the wild, and to undermine market traceability via mislabelling. In this study, we tested single nucleotide polymorphism (SNP) markers, recently developed to assess *H. gammarus* population structure^28,29^, for their application in discerning the two *Homarus* species and their hybrids, including their utility with pre-cooked material, with the aim of developing universal and accurate molecular tools for species and hybrid assignment.

## Methods

Clawed lobster tissues were all stored in 95–100% ethanol at −20 °C between sampling and DNA extraction. Tissue samples obtained were as follows:

- pleopods of 20 cooked *H. americanus*, described as originating from Maine, USA, sourced frozen from a UK-based seafood importer and sampled in 2019.
- pleopods of 30 cooked *H. gammarus*, originating from Cornwall, UK, sourced frozen from UK-based seafood suppliers and sampled in 2019.
- thirty whole zoeal stage I larvae, expected *H. americanus x gammarus* hybrids, hatched and sampled at Kristineberg Marine Research Station in Fiskebäckskil, western Sweden, in May and June 2017 from an ovigerous female *H. americanus* (92 mm carapace length) caught near Bornö Island in Gullmar fjord in October 2016. Siblings from this clutch had previously been reported as hybrids using three unpublished microsatellites (^20^, using the method in^23^).
- pereiopods of 18 live egg-bearing female *H. americanus*, comprising three individuals from each of six locations encompassing most of the species’ range (New Brunswick, Newfoundland, Prince Edward Island and Nova Scotia in Canada, and Massachusetts and New Hampshire in the USA). Tissues were collected by collaborating researchers.

Extraction of genomic DNA from these tissues was conducted using the salting-out technique of Jenkins *et al*.^28^. DNA yields were assessed using a Nanodrop One spectrophotometer and standardised to concentrations of 50–100 ng/μl, before electrophoresis on 1% agarose gels to check fragment quality. As expected, DNA from cooked samples frequently evidenced degradation via electrophoresis. Genotyping and scoring of the 96 SNP loci of Jenkins *et al*.^28^ was carried out on the Fluidigm EP1 system, following the protocols outlined in Jenkins *et al*.^29^. Two separate genotyping runs were conducted on all samples from cooked lobsters and expected hybrids to test repeatability of results. To provide reference samples and test the potential occurrence of hybrids among wild European lobster populations, these new data were added to a data set comprised of 1,278 *H. gammarus* genotyped by Jenkins *et al*.^29^. Quality control and filtering of genotype data followed the protocols outlined by Jenkins *et al*.^29^, except that the permissible missing data threshold was relaxed from 20% to 40% to ensure retention of three cooked samples that failed the stricter criteria.

**Figure 1:**
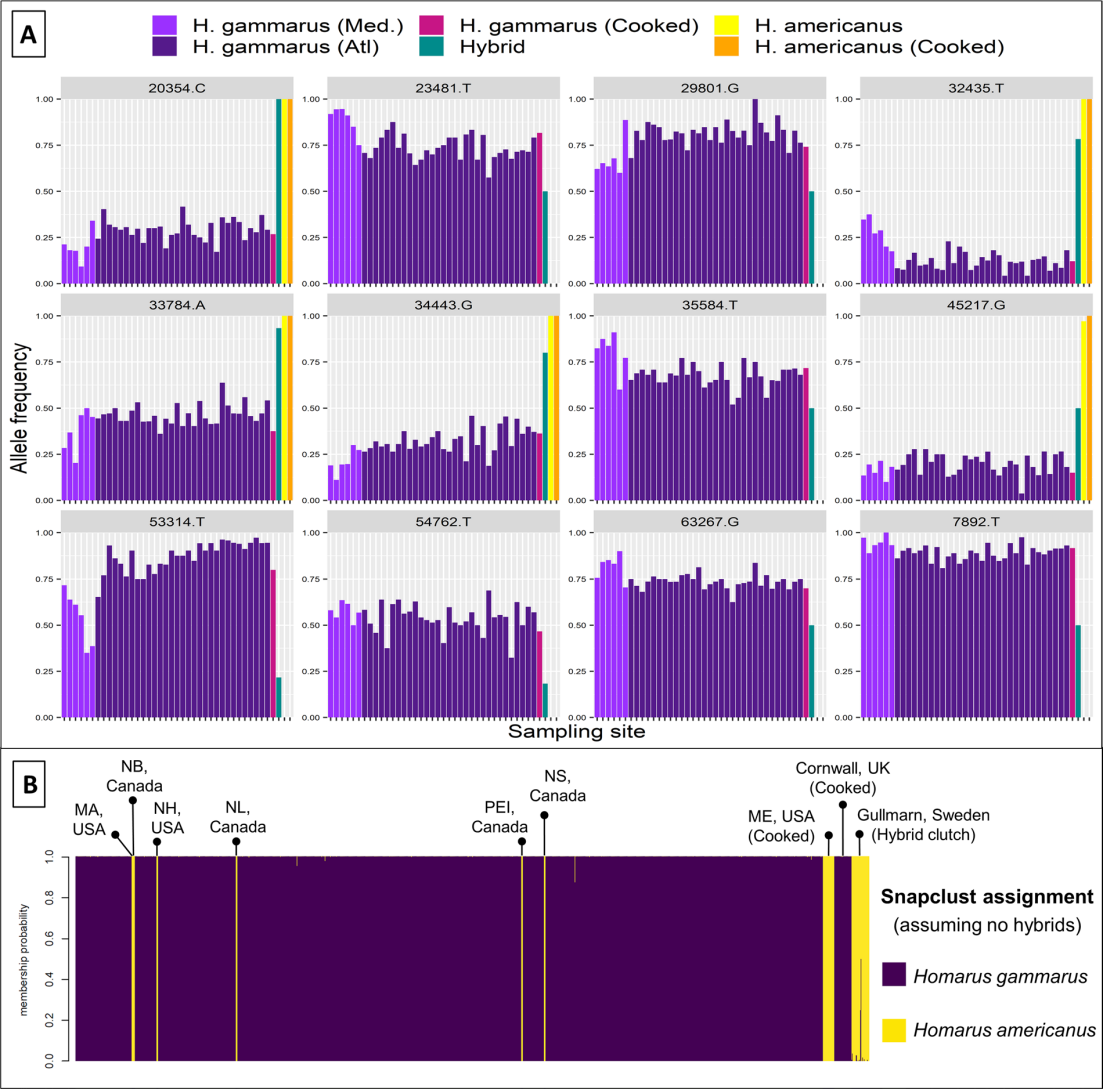
Plots of allele frequencies (**A**) and *snapclust* membership assignments to species level (**B**) for all 1,376 individuals genotyped at a subset of the 12 most informative SNP loci. In **A**, each vertical bar represents the mean allele frequency of a population sample for subset loci. Loci are named above each plot, with sample cohort colour coding at top. In **B**, individuals (arranged horizontally by sampling groups) are displayed as vertical bands, the colours of which denote their proportional membership assignments. Without assuming hybridisation, *snapclust* assigns all hybrids as majority *H. americanus*.

Genetic divergence was initially explored using discriminant analysis of principal components (DAPC), executed in the adegenet v2.1.1 package^30^ in R^31^. Cross-validation to select the optimal number of principal components to retain was conducted using the *xvalDapc* function in adegenet. Two analyses were used to test the assignment of species and the detection of hybrids. Firstly, adegenet was used to run *snapclust*^32^ which uses the Expectation–Maximization algorithm to calculate maximum-likelihood estimations of genetic clustering and admixture. The number of expected genetic clusters (*k*) was set to 2, with explicit modelling for the presence of hybrids between these two clusters (*hybrids* = TRUE) and all other parameters run at default. Secondly, the program StrAuto v1.0^33^ was used for parallel processing of the Bayesian clustering algorithm *STRUCTURE* v2.3.4^34^, implemented across 10,000 MCMC repetitions following a burn-in of 10,000, assuming an admixture model but without prior sampling information (*locprior* off) and all other parameters run at default. *STRUCTURE* run replicates were merged using *CLUMPP* v1.1.2^35^, and the optimal number of genetic groups (*K*) was defined using Δ*K*^36^ and mean L(*K*)^34^ with the pophelper v2.2.5.1 package^37^ in R. In addition, to test the performance of a reduced marker panel that could facilitate cheaper genotyping, the DAPC and *snapclust* analyses were repeated with a subset of the 12 most informative SNPs, as selected by DAPC loading contributions and inspection of allele frequencies (Fig. 1A). For this subset of loci, *snapclust* was run as above, both with and without expectations of hybrids being present.

## Results

As per Jenkins *et al*.^29^, filtering of the full 96-SNP data set removed 17 SNPs, leaving a panel of 79 SNPs (Supplementary Table S1), across which the mean level of missing data for all new samples was 3.0%. Among sample types, the mean level of missing data was 3.0% for hybrid larvae, 3.8% for cooked lobster samples, and 1.0% for live-sampled *H. americanus*. Aside from disparities caused by missing data, only 25 allele scores (0.4%) were mismatched between genotyping runs, all but three of which were attributed to inconsistent scoring at one locus.

**Figure 2:**
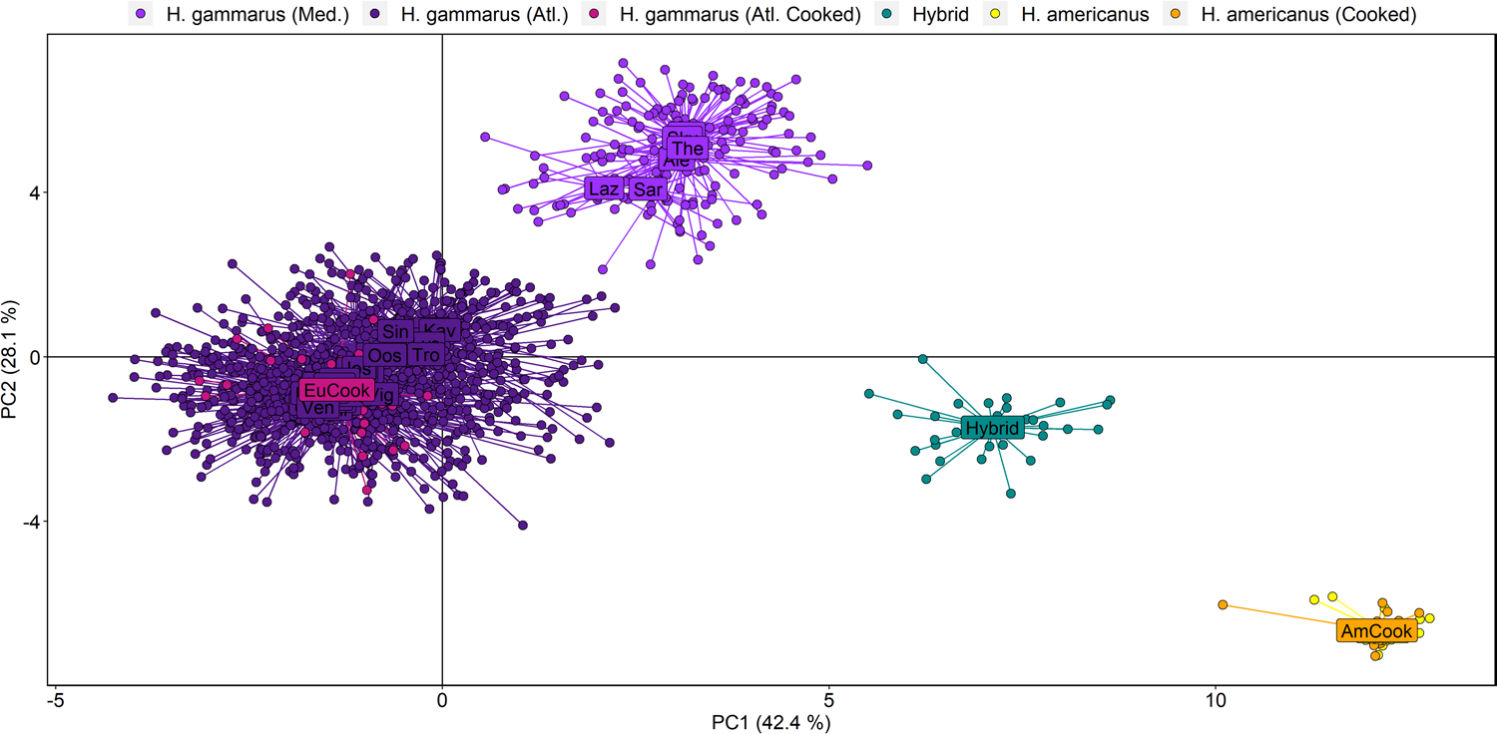
DAPC plot of principal component positions of individuals (dots) and sample means (labels), both coloured as per the key at the head of the figure, for the full array of 79 SNPs (after quality control filtering). Sample labels are 3-letter coded as per Jenkins *et al*.^29^, except new samples (6-letter coded).

Of the 79 SNP loci known to be highly polymorphic in *H. gammarus*^29^, only 12 were polymorphic across all *H. americanus* samples (*n* = 38), with 45 polymorphic among the clutch of hybrid siblings (*n* = 30). All except one of the 12-SNP subset were monomorphic in *H. americanus*, and the single locus to show heterozygosity did so in only one individual (Fig. 1A). DAPC showed clear and extensive separation of the two *Homarus* species across all 79 SNPs, with hybrids clustering together in a discrete group between these clades (Fig. 2). Cooked individuals clustered with the correct species clade, while there was also segregation between *H. gammarus* of Atlantic and Mediterranean origins, as demonstrated by Jenkins *et al*.^29^.

**Figure 3:**
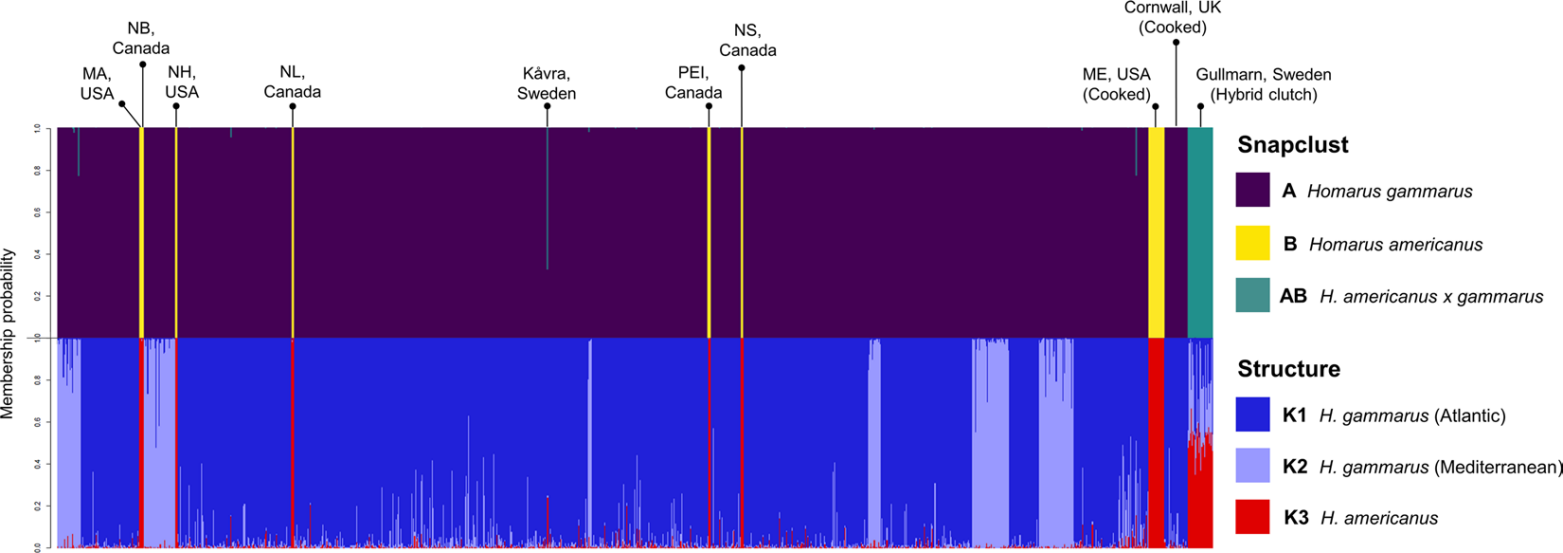
Plot of membership probabilities among all lobsters genotyped at the full array of 79 SNPs, as analysed by *snapclust* (upper) and *STRUCTURE* (lower). Individuals are displayed as vertical bands with colours denoting proportional membership assignments. Sample cohorts are grouped horizontally, with origin locations of sample groups or individuals of particular interest annotated above the *snapclust* plot.

Using all 79 SNPs, s*napclust* successfully detected all individuals of *H. americanus* and hybrid lineages (Fig. 2; Supplementary Fig. S1), with membership probabilities >0.999 (Supplementary Table S2). All cooked lobsters also assigned to the correct species at probabilities >0.999. Only one of the 1,308 assumed European lobsters—an individual from Kåvra in Sweden—was assigned majority hybrid membership by *snapclust* (to a component of 68%), and was also allocated a pronounced *H. americanus* component (~25%) using *STRUCTURE* (Fig. 3). Analysis of *STRUCTURE* outputs confirmed that *K* = 3 was the best-supported number of clades, although *STRUCTURE* placed greater emphasis on the intraspecific differentiation between *H. gammarus* of Atlantic and Mediterranean origins, so did not allocate a specific cluster to hybrids. Nevertheless, all hybrid individuals were still differentiated, with clade composition defined as approximately half to the *H. americanus* cluster, and half among the two *H. gammarus* clusters (Fig. 3).

Using only genotypes from the 12-SNP subset, *snapclust* assigned all *H. gammarus* and *H. americanus* (including cooked samples) to the correct species clade when run without assumptions of hybridisation (Fig. 1B; Supplementary Fig. S1). Only four *H. gammarus* individuals were assigned to probabilities <0.995, and only one of these was <0.95 (Supplementary Table S2). Notably, majority *H. americanus* composition was also assigned to all known hybrids via this analysis (Fig. 1B). When *snapclust* assumed the presence of hybridisation, known hybrids were detected and effective assignment of *H. americanus* was maintained, but 37 *H. gammarus* individuals (2.8% of the total) were incorrectly assigned with a majority component to the hybrid clade. These results were supported by DAPC, which showed little separation between some outlying *H. gammarus* individuals and the hybrid cluster using just 12 SNPs (Supplementary Fig. S2).

## Discussion

Using 79 SNPs originally developed to investigate intraspecific genetic differentiation among *H. gammarus* populations, we were instead able to highlight interspecific differences between *H. gammarus*, its congener *H. americanus*, and hybrids of the two species. Effective determination of both species and their hybrids were confirmed with the full 79-SNP panel using the *snapclust* software. Robust assignment was maintained using a subset of the 12 most powerful SNPs when assumptions of hybridisation were omitted; all individuals of both pure species were assigned correctly, and the introduced genetic component of hybrids was still detected, such that hybrids were assigned as *H. americanus* rather than *H. gammarus*. These biallelic loci can define species in *Homarus* because, while highly polymorphic in *H. gammarus*, the majority (85%) were monomorphic in the 38 *H. americanus* individuals we tested.

Our results show that these SNP loci present useful genetic tools for accurate taxonomic determination of *Homarus* lobsters. A subset of just 12 SNPs discriminates *H. americanus* from *H. gammarus*, facilitating the use of a genotyping chip such as the Fluidigm Flex Six Genotyping IFC, which can screen 72 individuals across 12 SNPs, but which can be loaded in six discrete sections of only 12 individuals at a time. This technology is therefore useful for processing small sample runs quickly and relatively cheaply (~£4 per individual), so is well suited to applications of routine screening or opportunistic checks of specimens caught and/or sold in Europe which are suspected to be introduced and/or mis-sold *H. americanus*. We have also demonstrated that this method can successfully genotype and determine species of pre-cooked lobsters, which few consumers or industry personnel could identify visually, even when sold whole. In practical terms, this tool could be used to screen for the genetic signature of *H. americanus* among putatively European lobsters captured or marketed in Europe. Although this 12-SNP tool lacks the resolution to reliably identify hybrids without generating false positives among pure *H. gammarus* specimens, it could still be useful in identifying samples warranting further analysis with the full SNP panel to test for hybridisation, since all the hybrids we tested assigned as *H. americanus* when analytical expectations were for the two pure species only. If necessary, *Homarus* hybrids could then be distinguished from specimens of the two pure species using all 79 SNPs.

All 30 *H. americanus x gammarus* offspring from Sweden were assigned hybrid status by *snapclust* using our 79-SNP tool. Only one assumed *H. gammarus* (out of 1,306) assigned as a majority hybrid by *snpaclust*. This individual (‘Kav26’) was a 103 mm CL male sampled in 2007 from the Kåvra lobster reserve in western Sweden^38^, approximately 20 km from the capture location of the introduced *H. americanus* female which carried the hybrid larval clutch tested in this study, and in an area which has experienced frequent introductions of American lobsters in recent years^20^. As such, although it seems plausible that this individual’s assignment as a hybrid represents an anomalous false positive, we cannot rule out the possibility that this specimen had some *H. americanus* ancestry via interspecific breeding; that this individual originated from a designated conservation reserve may warrant further investigation. Our study includes only one hybrid sibling cohort, so it would be beneficial to confirm the power of this 79-SNP tool on other suspected hybrid clutches, although the extent of interspecific differentiation we revealed suggests it should be universally applicable for identifying first-generation *Homarus* crosses.

Currently, it is difficult to ascertain the threat posed to European lobster stocks from hybridisation with *H. americanus*, since conflicting information exists as to both the likelihood of interspecific mating and the fertility of resultant hybrids. Talbot *et al*.^21^ found that the spermatophores of captive-reared hybrid males lacked sperm, suggesting that they were infertile, but Kitaka (*pers comm*., as cited by^22^) achieved interspecific mating in captivity and asserted that both sexes of the resultant hybrid offspring were fertile. Fertile hybrids enable backcrossing and pervasive population-level introgression, although hybridisation is still a conservation threat even where hybrids are infertile, since it represents wasted reproductive effort^5,6^. Mate-choice of *H. gammarus* females is to favour conspecific males regardless of their size and dominance status^22^, so the discovery of female *H. americanus* bearing hybrid clutches in the wild in Europe suggests that either American females more readily mate interspecifically than their European counterparts, or that behavioural barriers are not sufficient to prevent interspecific mating where no conspecific mate can be found. Accordingly, we recommend investigation of the propensity for interspecific mating, between both sexes of both species, and of the fertility status of hybrids of both sexes.

The recent motion from some European scientists and regulators to take a conservative and proactive approach to the threats posed by introduced *H. americanus* by outlawing its live-trade was met with robust resistance from advocates of commerce initially valued at US$260 million a year to North American exporters^12,13^. Indeed, instead of restricting live-trade, a newly-adopted EU trade agreement eliminates 6–20% tariffs on Canadian exporters, providing them with duty-free access to European markets^39^. Although existing regulations prohibit the release of non-native lobsters across most of Europe, this has not been sufficient to prevent increasing numbers of both inadvertent and deliberate introductions of *H. americanus*, heightening the likelihood of it becoming permanently established and/or damage from hybridisation^14–16^. In the wake of our confirmation that interspecific crossbreeding between native and introduced lobsters has occurred in the wild, greater effort should be made to educate regulators, commercial traders and the general public as to the potential conservation threats posed by hybridisation between endemic and introduced lobsters.

Although clear opportunity exists to profit from the mislabelling of cheaper *H. americanus* as the more expensive *H. gammarus*, the extent to which erroneous species designation—whether inadvertent or fraudulent—currently impacts lobster supply chains is unknown, as is the extent to which hybridisation with introduced *H. americanus* may be impacting wild European lobster populations. Such investigations can now be implemented using these SNP resources. The cost of genetic techniques is often cited to justify why they are not more widely used to enforce regulatory standards in the seafood sector; in practice, however, the expense of molecular testing is typically far exceeded by financial penalties levied against uncovered incidences of malpractice^40^. Overall, the approaches we have tested offer more powerful assignment of species and hybridisation in clawed lobsters than existing methods based on allozymes^41^, random amplified polymorphic DNA^42^ or microsatellites^23^, and enable accurate and rapid sample processing via high-throughput SNP genotyping technology. These assignment techniques can be applied in forensic assessments to address the extent that introduced *H. americanus* threaten populations of *H. gammarus* via hybridisation and invasive introgression, and whether fraudulent mislabelling affects clawed lobster seafood products in Europe.

## Supplementary information

Supplementary Information.

Supplementary Table S1 .

Supplementary Table S2.
