# Supplementary material for: Crossing the pond: genetic assignment detects lobster hybridisation
Source: Sci Rep. 2020 May 8;10:7781. doi: 10.1038/s41598-020-64692-z (PMC7210874; doi:10.1038/s41598-020-64692-z)
Supplement: Supplementary file 1 — Supplementary Information. [file 41598_2020_64692_MOESM1_ESM.pdf]

# Crossing the pond: genetic assignment detects lobster hybridisation

Charlie D. Ellis, Tom L. Jenkins, Linda Svanberg, Susanne P. Eriksson & Jamie R. Stevens

**Scientific Reports (2020)**

Supplementary Information – Figures S1 & S2.

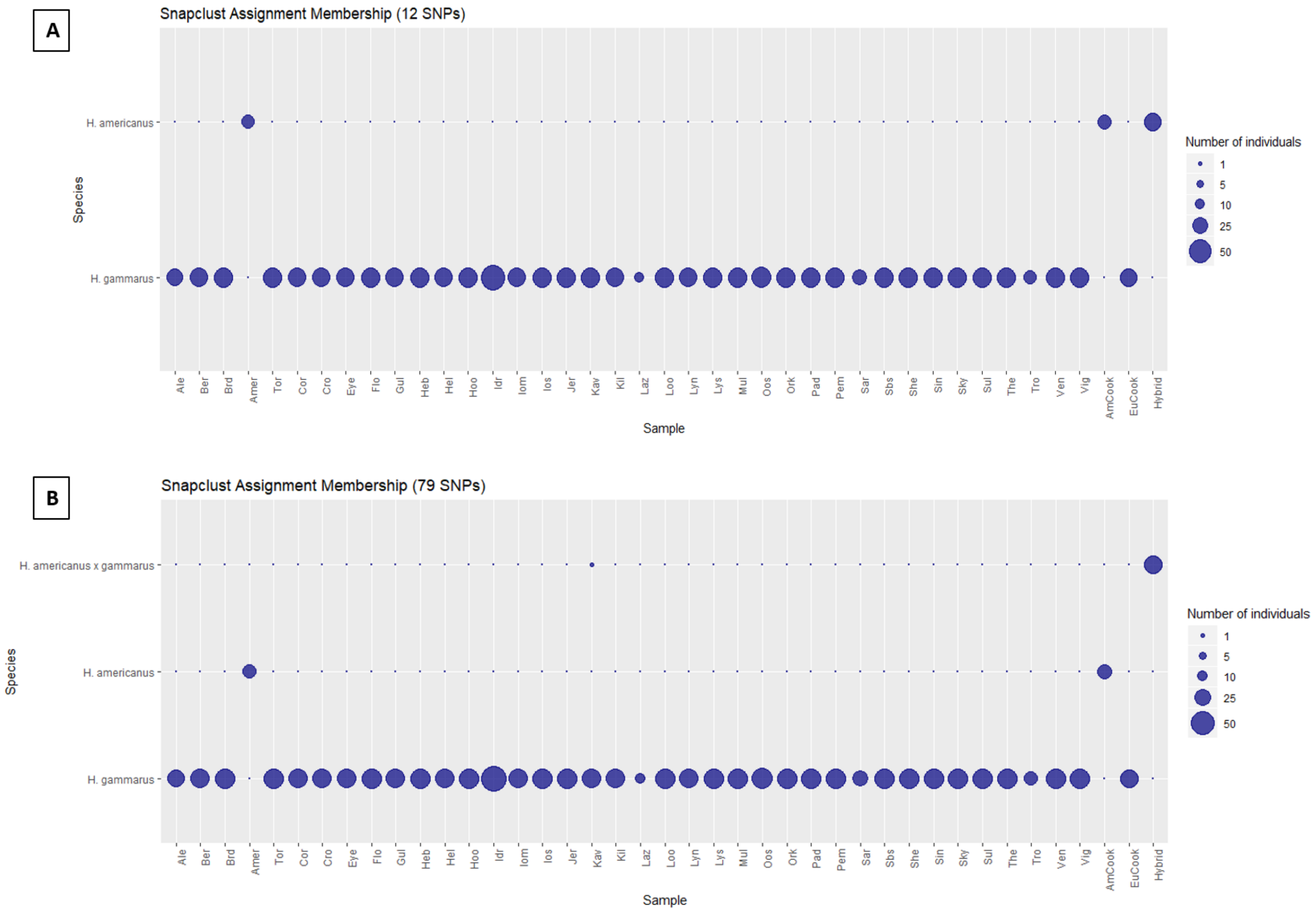


**Supplementary Figure S1**: Plots showing the frequency of majority assignment to each species clade by sample groups, as defined by *snapclust* for both the subset panel of 12 SNPs (**A**, upper, assuming absence of hybridisation) and the full panel of 79 SNPs (**B**, lower, assuming presence of hybridisation). Sample groups are coded on the x-axis as per Jenkins *et al* (2019)*, except for the new samples added to this study: 'Amer' (18x live *H. americanus*); 'AmCook' (20x cooked *H. americanus*); 'EuCook' (30x cooked *H. gammarus*), and; 'Hybrid' (30x *H. americanus x gammarus* larval siblings).

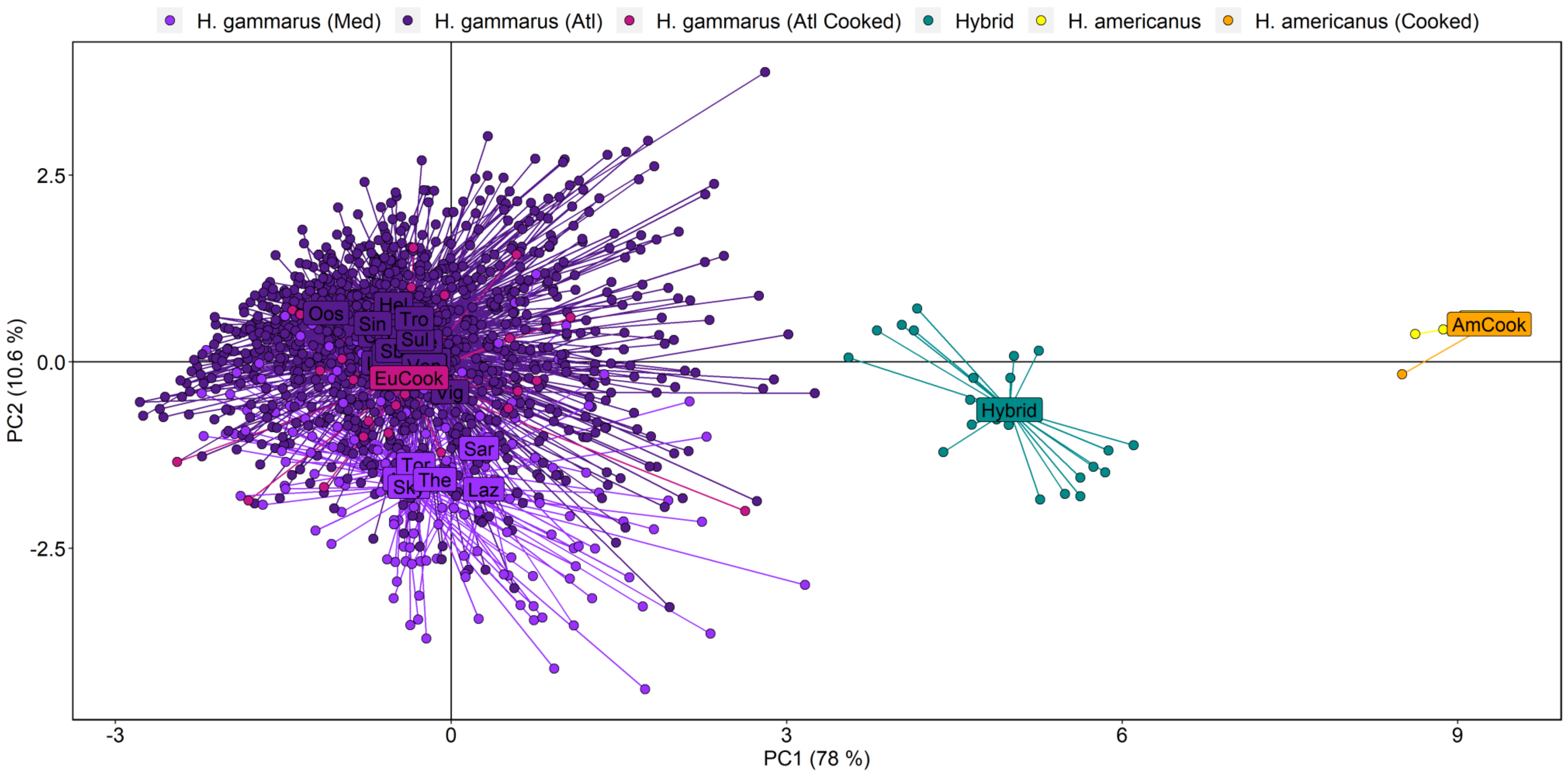


**Supplementary Figure S2**: DAPC plot of principal component positions of individuals (dots) and sample means (labels), both coloured as per the key at the head of the figure, for the subset array of 12 SNPs (after quality control filtering). Sample labels are 3-letter coded as per Jenkins *et al* (2019)*, except new samples (6-letter coded).


* = Jenkins, T.L., Ellis, C.D., Triantafyllidis, A. & Stevens, J.R. Single nucleotide polymorphisms reveal a genetic cline across the northeast Atlantic and enable powerful population assignment in the European lobster. *Evol. Apps.* **12**, 1881-1889 (2019).
